# Haringey Rehabilitation Pathway

**DOI:** 10.1192/bjo.2026.11650

**Published:** 2026-06-30

**Authors:** khaver bashir, HANNAH CAMPLING, Kerby Francis, Daisy Mckenna, Tehmina Iqbal

**Affiliations:** North London Foundation Trust, London, United Kingdom

## Abstract

**Aims:**

We wanted to develop a dedicated pathway to support people through their rehab journey from the start of their inpatient admission until 18 months post discharge. The overarching aim of this pathway is to improve the service user journey whilst saving the trust money at the same time.

**Methods:**

A thorough service evaluation across the five boroughs within North London Foundation Trust (NLFT) in order to map out the current processes in place and discrepancies between services.

Literature review of current practices in rehabilitation psychiatry across the country, cross referenced with NICE guidelines to establish the best evidence-based pathway.

Most importantly qualitative research from interviews with service users, carers and staff in order to develop a co-produced pathway that is driven by the needs of those we are trying to support.

The above methods were used to co-produce a pilot rehabilitation pathway in Haringey which began in May 2024.

**Results:**

Our pilot project has reduced the number of rehab bed days by 2695 (projected for end of May 2026), which is a reduced Integrated Care Board (ICB) spend of £1540,000 (based on average cost of rehab bed of £4000/week).

Our re-admission shows 302 days saved at a cost saving of £172,571.

Our qualitative data shows service users under the pathway are able to live more independently, are engaging with our Occupational Therapy (OT) and psychology groups, have reductions in their package of care needs and as a result of the reduction in out of area beds we are able to deliver our care, closer to home.

**Conclusion:**

The Haringey Rehabilitation Pathway is an excellent example of a co-produced pathway that addresses the needs identified from a robust service evaluation.

The outcomes of the pilot pathway show considerable financial savings to the trust and ICB. There has been a dramatic reduction in the number of rehabilitation bed days, out of area placements and readmission rates and our service users feedback is proof that this pathway is supportive and enables independence by focussing on relationship building and development of life skills.

Future developments include rolling out our pathway across the whole trust.

